# Mepolizumab does not alter the blood basophil count in severe asthma

**DOI:** 10.1111/all.13879

**Published:** 2019-06-28

**Authors:** Adam Kelvin Alec Wright, Sarah Diver, Jamie McCarthy, Andrew Marvin, Marcia Soares, Tracy Thornton, Michelle Bourne, Michelle Craner, Helen Evans, Sarah Edwards, Sarah Glover, Liesl Carr, Sarah Parker, Salman Siddiqui, David Cousins, Chris Brightling

**Affiliations:** ^1^ Department of Respiratory Sciences University of Leicester Leicester UK; ^2^ National Institute for Health Research, Leicester Respiratory Biomedical Research Centre University Hospitals of Leicester NHS Trust Leicester UK; ^3^ Pathology Services University Hospitals of Leicester NHS Trust Leicester UK; ^4^ Respiratory Medicine University Hospitals of Leicester NHS Trust, Glenfield Hospital Leicester UK

##  

To the Editor:

Mepolizumab (anti‐IL‐5) depletes blood and airway eosinophils, and, clinically*,* allows down‐titration of oral corticosteroid and a reduction in the frequency of eosinophil‐dependent exacerbations.^1^ Basophils also express IL‐5Rα, participate in T2‐mediated inflammatory pathways^2^ and have been associated with exacerbation frequency.^3^ Whilst basophil progenitors are unlikely to depend on IL‐5 for development,^4^ blood basophil counts measured in routine clinical laboratories suggest they decrease following mepolizumab treatment.^5^, ^6^, ^7^, ^8^

Our primary objective was to determine whether anti‐IL‐5 monoclonal antibody treatment reduces blood basophil levels as an additional potential efficacy mechanism. To achieve this, we measured blood basophils, eosinophils and other type 2 inflammatory cells, before and after 16 weeks of mepolizumab (“Nucala,” GlaxoSmithKline) by flow cytometry. Patient eligibility criteria are in the online supplement and the study schedule in Figure S1.

Blood samples were obtained from 26 severe asthma subjects, attending a difficult asthma clinic at a single UK centre, at baseline and following a median (IQR) of 16 (16‐17) weeks of mepolizumab, administered as a 100 mg subcutaneous injection every 4 weeks. In 2 cases, it was not possible to obtain post‐treatment samples (n = 1, withdrew consent; n = 1, discontinued), totalling 24 (Figure S1 and Table S1). We also recruited 15 nonasthmatic healthy controls (Table S1) to obtain samples at parallel time points but without an intervention (Figure S1). Flow cytometric measurements were compared with data derived through the routine pathology service, which utilizes an ADVIA 2120/2120i analyser (Siemens, UK). A detailed description of the methodology for both approaches is described in the online supplement, and for flow cytometry, the gating strategy is shown in Figure S2. Our criteria for identifying cell subsets were as follows: eosinophils (CD45^+^CD3^‐^CD193^+^CD294^+^SSC^hi^CD123^−/+^), basophils (CD45^+^CD3^‐^CD193^+^CD294^+^SSC^lo^CD123^+^), cTH2 and peTH2 (both CD4^+^CD294^+^ but CD161^−^ or ^+^, respectively), cTC2 (CD8^+^CD294^+^) and ILC2s (Lineage^−^CD294^+^CD161^+^).

For methodological comparisons, data from asthma and healthy subjects (n = 39) were pooled. A good correlation was observed between flow cytometry and the ADVIA 2120i for total cells (*r* ^2^ = 0.24, *P* = 0.0014) and eosinophils (*r* ^2^ = 0.75, *P* < 0.0001), but not basophils (*r* ^2^ = 0.06, *P* = 0.13 Figure S3). In addition, the change in cell concentration between baseline and follow‐up showed a good correlation between the two analytical methods for eosinophils (*r* ^2^ = 0.72, *P* < 0.0001, n = 38) but not basophils (*r* ^2^ = 0.02, *P* = 0.39, n = 38).

As expected, following 16 weeks of mepolizumab, we observed a significant reduction in blood eosinophil concentration (flow cytometry [mean ± SD] −6442 ± 6852, ADVIA −20 688 ± 19 355 cells/100 µL, Figure 1A) and frequency (flow cytometry [mean ± SD] −1.2 ± 1.2% of total leukocytes, Figure S4A) compared with baseline levels for both methods. This decrease was not observed in our control group (mean ± SD 2475 ± 4148, 285 ± 6977 cells/100 µL and 0.70 ± 1.38%, respectively) (Figure 1A). Notably, the reduction in blood eosinophil levels following mepolizumab was related to baseline eosinophil levels (*r* ^2^ = 0.69, *P* < 0.0001, Figure S5).

**Figure 1:**
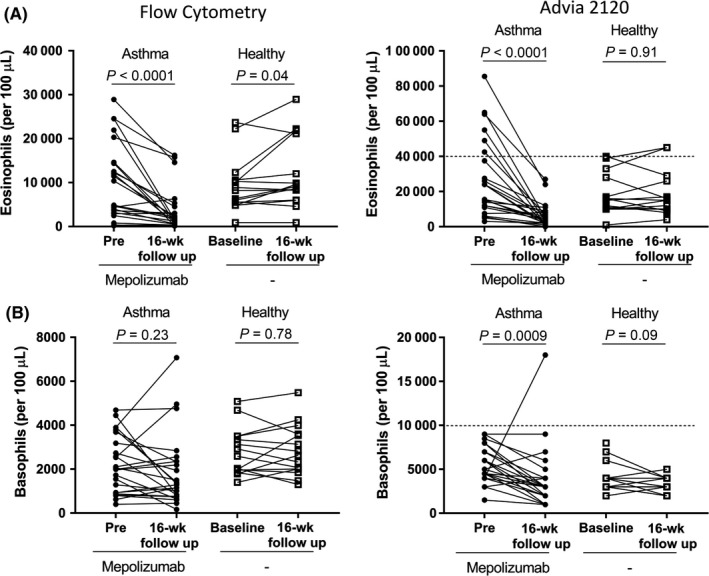
Eosinophil and basophils were measured per 100 µL blood using flow cytometry (A and B, left) and on an ADVIA 2120/2120i analyser (A and B, right). In the asthma group, the eosinophil and basophil concentration was measured before and after 16 wks of mepolizumab. In the healthy group, the eosinophil and basophil concentration was measured at baseline and 16 wks later. Within‐group comparisons were made with a Wilcoxon matched‐pairs test or a paired *t* test (healthy group: A, right and B, left). Dashed line indicates ADVIA 2120/2120i upper reference range

In contrast to eosinophils, basophil concentration and frequency at baseline were similar to that following 16 weeks of mepolizumab, when measured by flow cytometry (pre vs post [mean ± SD] 2232 ± 1309 vs 1873 ± 1647 cells/100 µL, *P* = 0.23, Figure 1B and 0.40 ± 0.19 vs 0.37 ± 0.27% of total leukocytes, *P* = 0.076, Figure S4B). Surprisingly, measurements obtained on the ADVIA 2120i suggested a statistically significant reduction in the basophil concentration following 16 weeks of mepolizumab (pre vs post [mean ± SD] 5521 ± 2003 vs 3792 ± 3623 cells/100 µL, *P* = 0.0009, Figure 1B). The mean ± SD reduction in basophil concentration of −1667 ± 3988 cells/100 µL in asthma was significantly greater compared with the change observed in the control group (vs −571 ± 1222, *P* = 0.011, Mann‐Whitney). In the healthy group, the concentration and frequency of basophils were similar when comparing baseline and follow‐up samples, regardless of analytical method (Figures 1B and S4).

These data suggest that basophil concentration and frequency, alongside other T2 inflammatory cells (Figure S6), are likely to be IL‐5/mepolizumab‐independent in severe asthma. However, our real‐world study was not sufficiently powered to detect small differences in relation to basophil concentration or frequency. A strength of our flow cytometric approach is that we have measured cell concentration as well as frequency, and also reported recently identified T2 cell subsets (eg peTH2 cells). Our data suggest there were also no indirect effects of mepolizumab on type‐2 polarised T cell or group 2 ILC concentration over this 16‐week time frame as indicated by others.^5^

Clinically, we observed a significant change in ACQ6 symptom score from a baseline of 2.9 ± 1.6 to 1.9 ± 1.3 at 16 weeks post‐treatment, which is a reduction of −0.92 (97.73% CI of −2 to −0.16, Wilcoxon matched pairs, *P* = 0.0085), corresponding to an improvement in symptoms above the minimal clinically important difference (MCID) threshold of −0.5. Since baseline eosinophil concentration was associated with basophils (*r* = 0.53, *P* = 0.0073), cTH2 (*r* = 0.59, *P* = 0.0023), peTH2 (*r* = 0.45, *P* = 0.026) and TC2 (*r* = 0.45, *P* = 0.026) cells but not total cells (*r* = 0.2, *P* = 0.35) or ILC2s (*r* = 0.2, *P* = 0.35), we examined their relationship to ACQ6 improvement (ΔACQ6). The baseline cellular parameters described above were not associated with ΔACQ6 (Figure 2, shown for eosinophils, cTH2 and peTH2 cells only). ΔACQ6 was also not associated with the Δ change in eosinophil (*r* ^2^ = 0.04, *P* = 0.36) or basophil (*r* ^2^ = 0.09, *P* = 0.15) levels post‐treatment, limiting the utility of these measurements as symptom response biomarkers.

**Figure 2:**
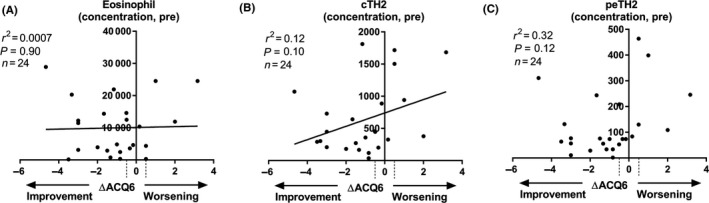
(A) Eosinophil, (B) cTH2 and (C) peTH2 concentration pre‐mepolizumab (y‐axis) plotted with the absolute change in ACQ6 score between baseline and 16 wks post‐mepolizumab (ΔACQ6, x‐axis). Linear regression (A, B) or Spearman (C) analysis was performed. Dashed lines on x‐axis represent the minimal clinically important difference (±0.5)

Of further interest was the effect of mepolizumab on eosinophil and basophil cell surface expression of the IL‐3 receptor α (CD123), and their relationship to ΔACQ6. IL‐3 is upregulated in the serum of poorly controlled asthmatic patients,^9^ and it can potentiate eosinophil chemotactic and degranulation responses. Recently, mepolizumab treatment, in the context of allergen challenge,^8^ was associated with reduced levels of IL‐3Rα mRNA and protein on circulating blood but not lung eosinophils.

Consistent with Kelly et al*,* we observed a decrease in eosinophil IL‐3Rα cell surface expression in response to mepolizumab in asthma (pre [mean ± SD] 415 ± 306 vs post 204 ± 192 GMFI, *P* < 0.0001, n = 21) and not in our healthy control group (baseline [mean ± SD] 587 ± 626 vs post 558 ± 535 GMFI, *P* = 0.32, n = 15) (example in Figure S2 and cumulative in S7). This represents a % decrease of −54 ± 25% in asthma compared with +9 ± 55% in the healthy group (mean ± SD, *P* < 0.0001, Mann‐Whitney test). We also noted that eosinophil expression of CRTH2 (CD294) was increased following mepolizumab (Figure S7); however, there was no relationship between the reduction in eosinophil IL‐3Rα expression and CRTH2 expression. Importantly, there was no relationship between changes in eosinophil IL‐3Ra or CRTH2 expression with ΔACQ6 in these patients. Furthermore, there were no mepolizumab‐dependent effects on eosinophil/basophil Siglec‐8, CD69 or IL‐5Rα expression (not shown), consistent with others^8^ or basophil IL‐3Rα (Figure S7),^.^and thus, these parameters were not examined for a relationship with ΔACQ6.

In summary, our flow cytometric data do not support a direct inhibitory effect of mepolizumab on basophil levels, and therefore, clinical benefit is likely to be independent of basophils. Our data suggest that the specificity and sensitivity of basophil detection on routine clinical analysers should be validated prior to reporting/interpreting basophil data in the context of an intervention. Our data do support others^8^ that mepolizumab reduces eosinophil, but not basophil, IL‐3Rα expression and, importantly, extends the applicability of this phenomenon to the “real‐world” scenario. However, neither changes in eosinophil levels nor changes in IL‐3Rα expression were associated with clinical efficacy determined by change in asthma control in this study, and thus, biological correlates of response to treatment require further study.

## FUNDING INFORMATION

This research was funded by Leicester Drug Discovery and Diagnostics (LD^3^) with financial contributions from MRC grant MC_PC_15045 and supported by the NIHR Leicester Biomedical Research Centre.

## References

[1] Russell R , Brightling C . Mepolizumab for the reduction of exacerbations in severe eosinophilic asthma. Expert Rev Respir Med. 2016;10(6):607‐617. 27058452 10.1080/17476348.2016.1176532

[2] Suzuki Y , Wakahara K , Nishio T , Ito S , Hasegawa Y . Airway basophils are increased and activated in eosinophilic asthma. Allergy 2017;72(10):1532–1539. 28474352 10.1111/all.13197

[3] Leffler J , Jones AC , Hollams EM , et al. Basophil counts in PBMC populations during childhood acute wheeze/asthma are associated with future exacerbations. J Allergy Clin Immunol. 2018;142(5):1639–1641. 30036597 10.1016/j.jaci.2018.07.003

[4] Mori Y , Iwasaki H , Kohno K , et al. Identification of the human eosinophil lineage‐committed progenitor: revision of phenotypic definition of the human common myeloid progenitor. J Exp Med. 2009;206(1):183–193. 19114669 10.1084/jem.20081756 PMC2626675

[5] Buttner C , Lun A , Splettstoesser T , Kunkel G , Renz H . Monoclonal anti‐interleukin‐5 treatment suppresses eosinophil but not T‐cell functions. Eur Respir J. 2003;21(5):799–803. 12765424 10.1183/09031936.03.00027302

[6] Flood‐Page P , Menzies‐Gow A , Phipps S , et al. Anti‐IL‐5 treatment reduces deposition of ECM proteins in the bronchial subepithelial basement membrane of mild atopic asthmatics. J Clin Invest. 2003;112(7):1029–1036. 14523040 10.1172/JCI17974 PMC198522

[7] Nair P , Pizzichini M , Kjarsgaard M , et al. Mepolizumab for prednisone‐dependent asthma with sputum Eosinophilia. N Engl J Med. 2009;360(10):985–993. 19264687 10.1056/NEJMoa0805435

[8] Kelly EA , Esnault S , Liu LY , et al. Mepolizumab attenuates airway eosinophil numbers, but not their functional phenotype, in asthma. Am J Respir Crit Care Med. 2017;196(11):1385–1395. 28862877 10.1164/rccm.201611-2234OC PMC5736971

[9] Patil SP , Wisnivesky JP , Busse PJ , Halm EA , Li XM . Detection of immunological biomarkers correlated with asthma control and quality of life measurements in sera from chronic asthmatic patients. Ann Allergy Asthma Immunol. 2011;106(3):205–213. 21354022 10.1016/j.anai.2010.11.019 PMC4648242

