# Supplementary figures and images for: Mepolizumab does not alter the blood basophil count in severe asthma
Source: Allergy. 2019 Jun 28;74(12):2488–90. doi: 10.1111/all.13879 (PMC6973167; doi:10.1111/all.13879)

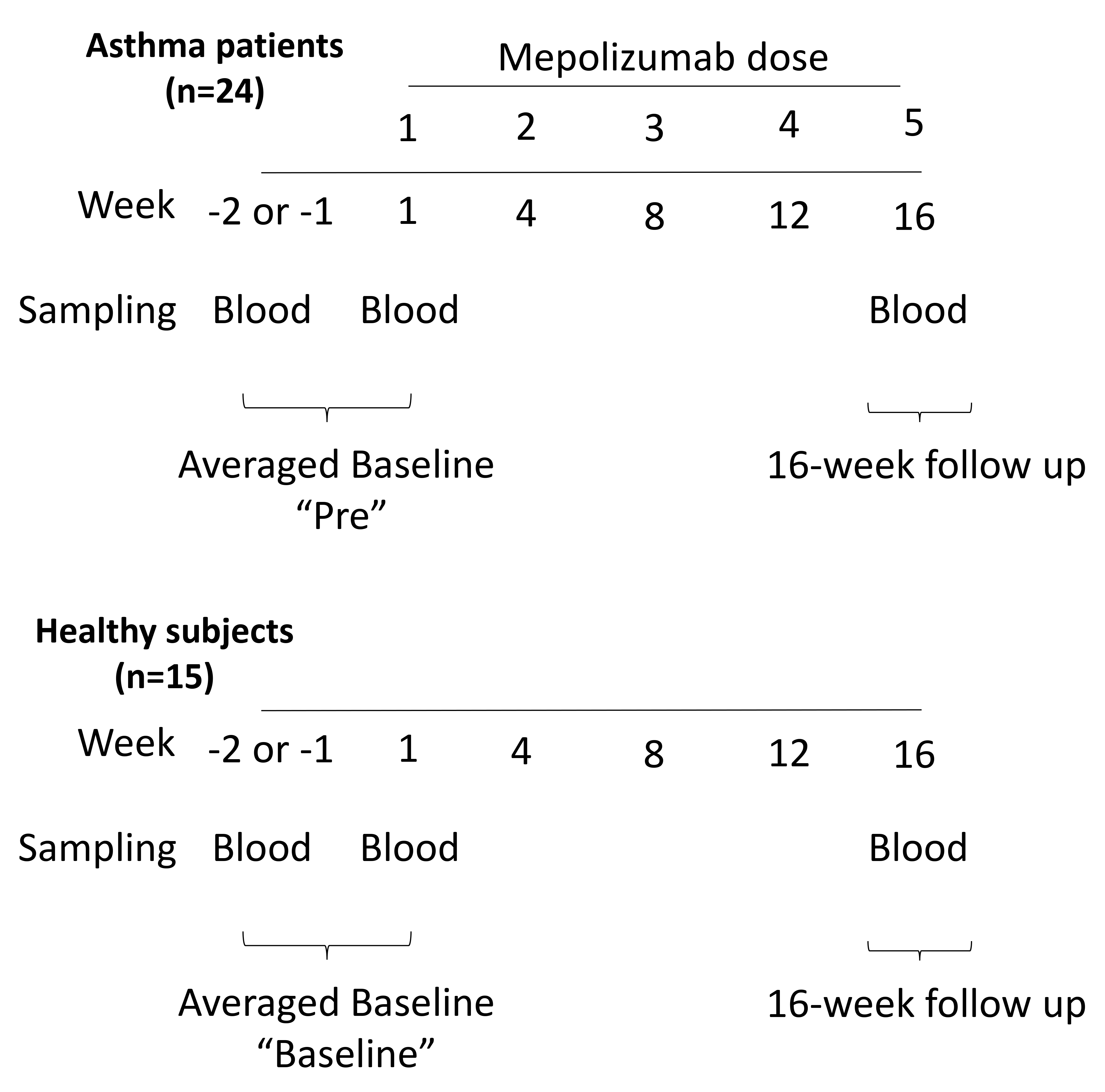

Supplement: Supplementary file 1 [file ALL-74-2488-s001.tif]

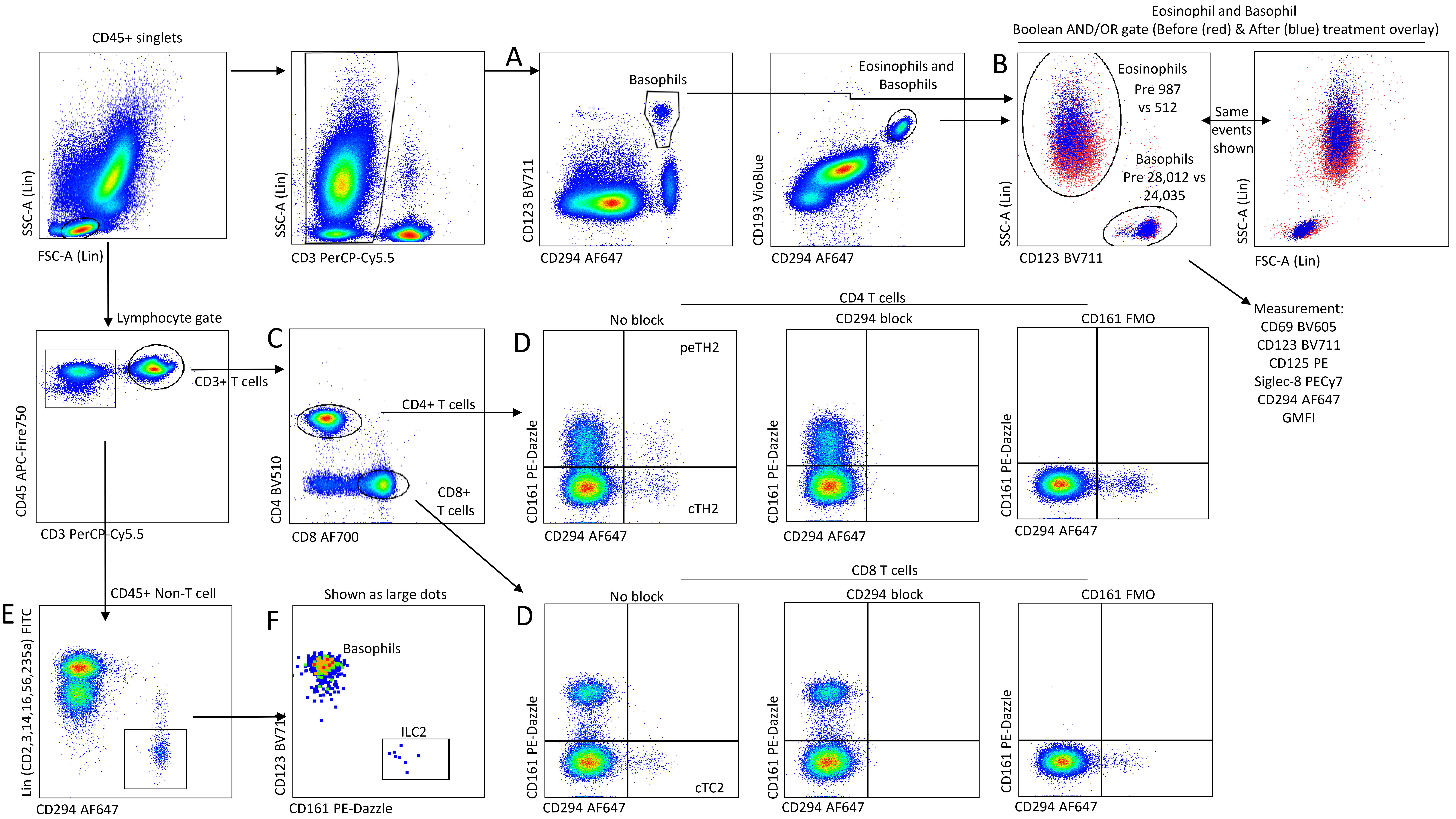

Supplement: Supplementary file 2 [file ALL-74-2488-s002.tif]

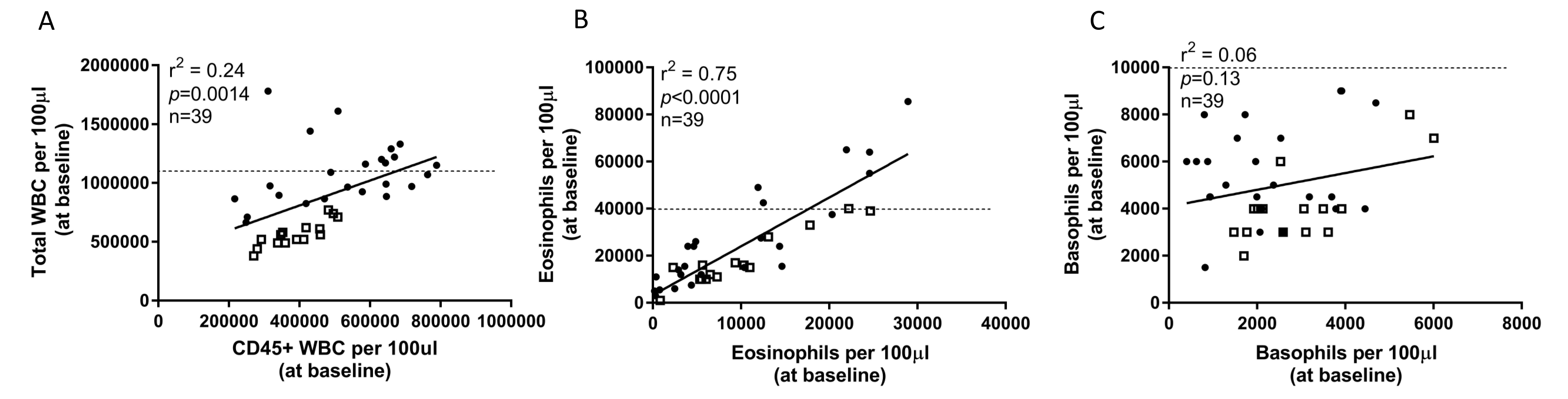

Supplement: Supplementary file 3 [file ALL-74-2488-s003.tif]

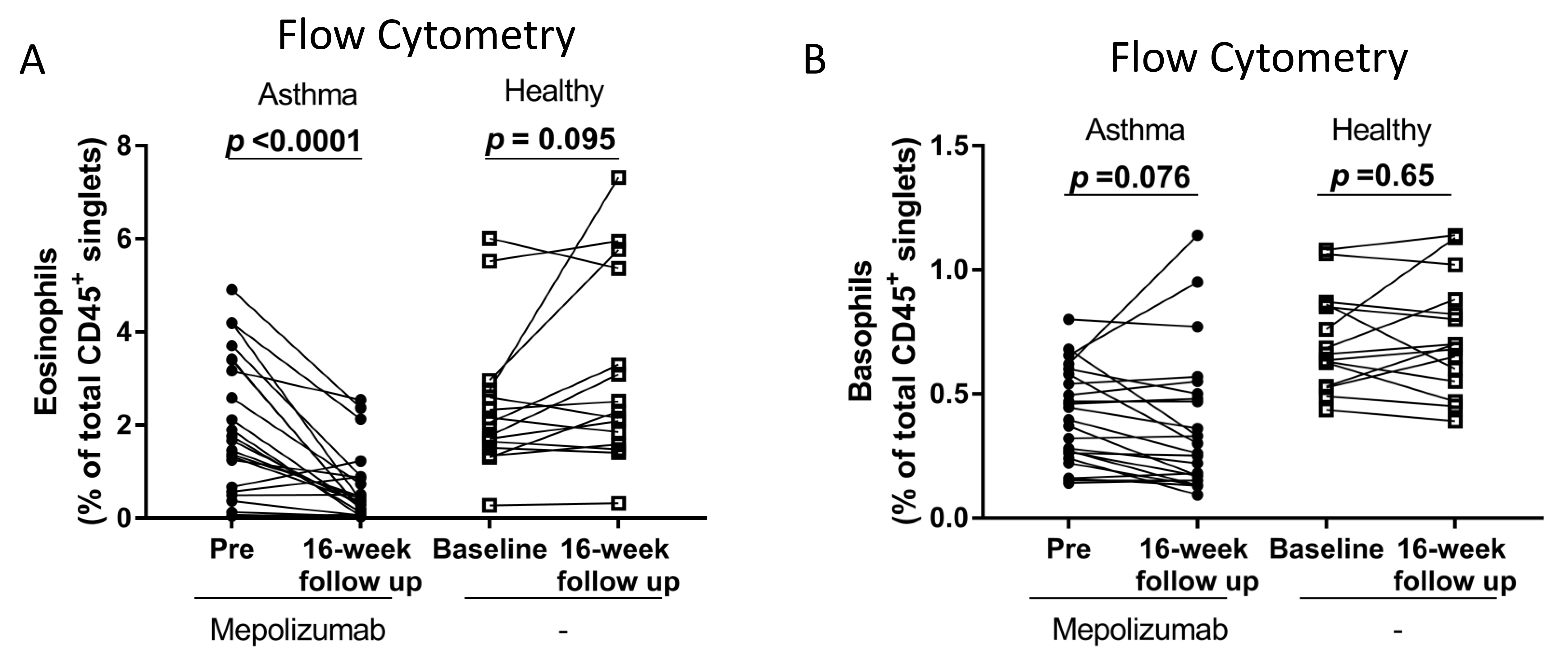

Supplement: Supplementary file 4 [file ALL-74-2488-s004.tif]

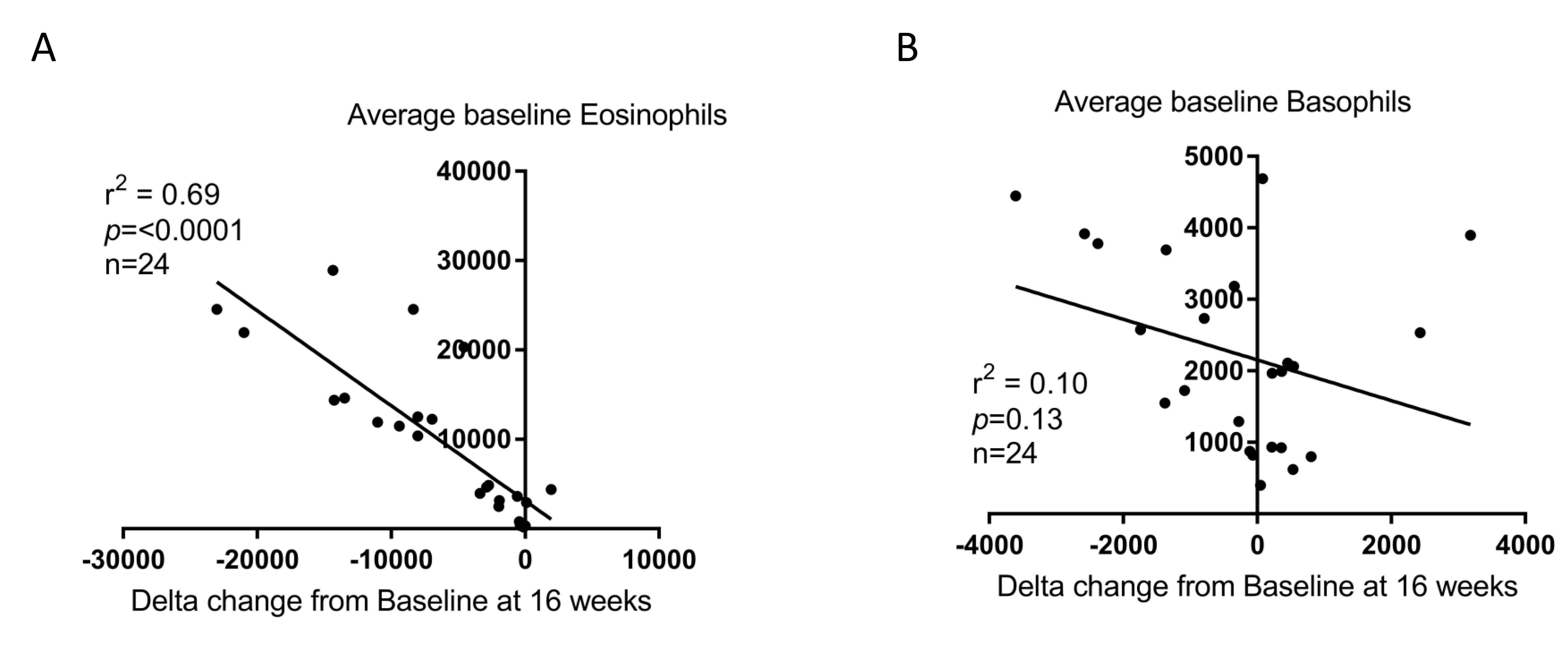

Supplement: Supplementary file 5 [file ALL-74-2488-s005.tif]

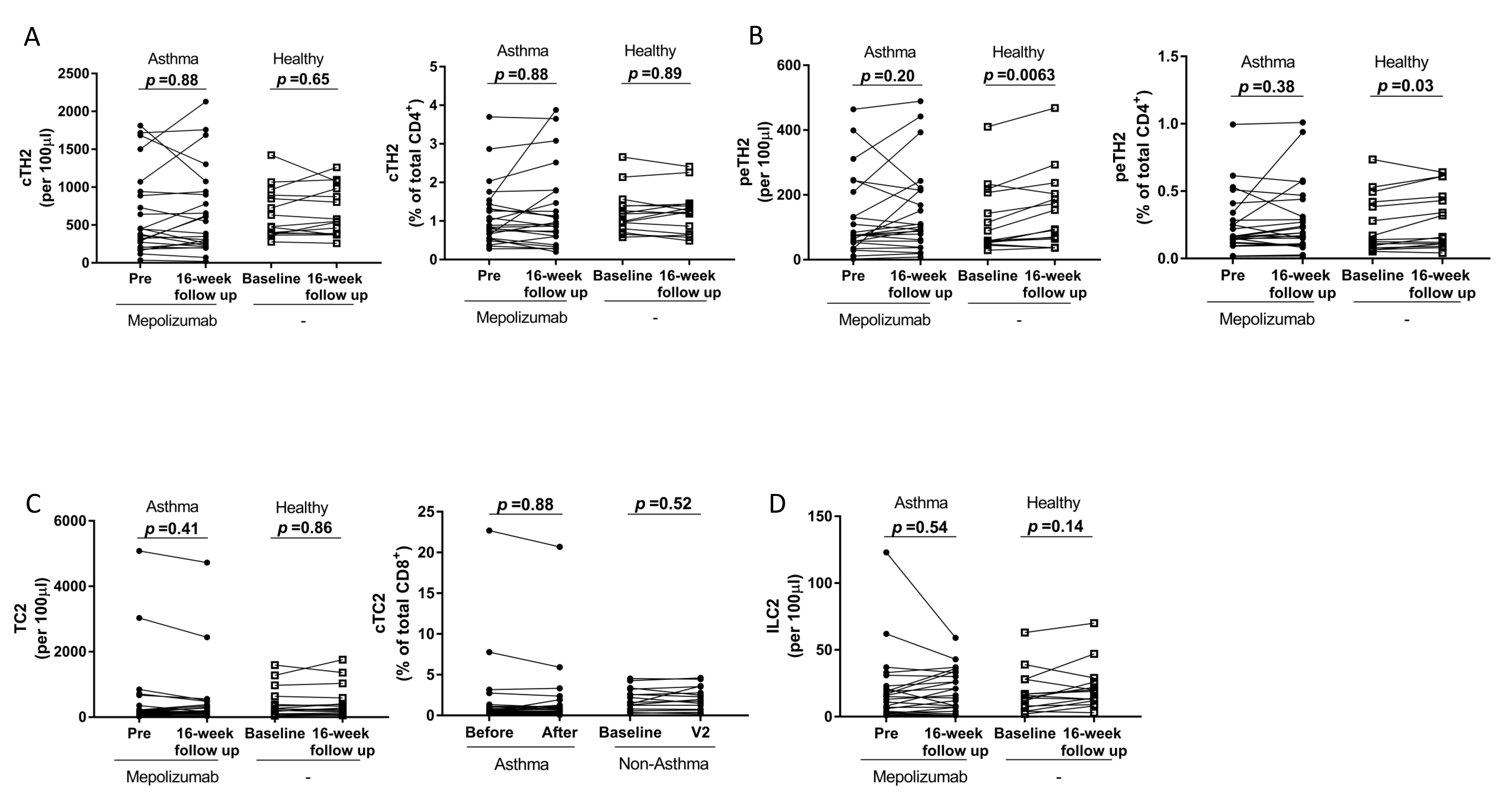

Supplement: Supplementary file 6 [file ALL-74-2488-s006.tif]

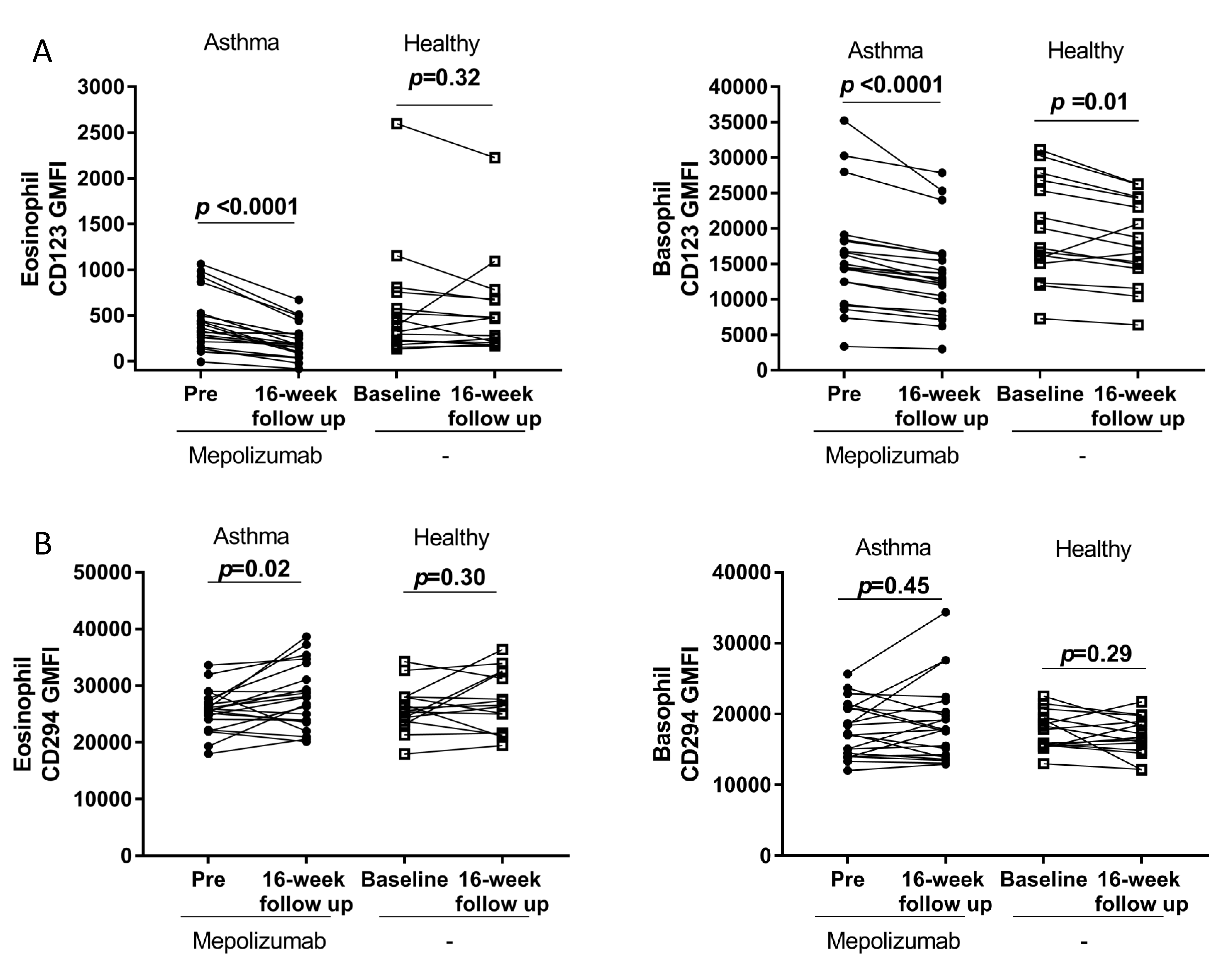

Supplement: Supplementary file 7 [file ALL-74-2488-s007.tif]
