# Supplementary material for: Mepolizumab does not alter the blood basophil count in severe asthma
Source: Allergy. 2019 Jun 28;74(12):2488–90. doi: 10.1111/all.13879 (PMC6973167; doi:10.1111/all.13879)
Supplement: Supplementary file 8 [file ALL-74-2488-s008.docx]

**Mepolizumab does not alter the blood basophil count in severe asthma**

Online Supplement

Methods

**Subjects**

We recruited 26 severe asthma patients that had been approved as meeting National Institute of Clinical Excellence (NICE) guidelines published on the 25^th^ January 2017 by our asthma multi-disciplinary team to receive 4-weekly 100mg sub cutaneous mepolizumab injections (“Nucala”, Glaxo-SmithKline) within a dedicated outpatient clinic (Figure E1). The criteria for mepolizumab eligibility are eosinophil levels >300 cells/µl at the time of assessment (or anytime in the past 12 months) *and* either more than 2 exacerbations requiring OCS (in the past 12 months) or is on at least 5mg per day of OCS over the past 6 months. If approved for mepolizumab, consent was obtained in accordance with Leicestershire and Rutland ethical approval (REC ref 08/H0406/189) at least 24 hours prior to the visit. Routine clinical measurements were obtained within the outpatient clinic, at baseline (Table E1), prior to the commencement of mepolizumab, and at 16-week review points (not shown). At recruitment, patients were obese (BMI 33 ± 9 Kg/m^2^), had a median [IQR] eosinophil level of 198 [112-412] cells/µl, moderate symptom scores (ACQ6 2.9±1.6, Total AQLQ 3.8±1.3), a median of 6 [2-9] exacerbations in the past 12 months, a median daily OCS dose of 15 [10-15] and a median inhaled steroid dose of 1600 [1600-1600]µg/day BDP equivalent. The post-treatment ACQ6 (mean±SD) was 1.86±1.33, which is a mean difference of -0.92 [97.73% CI of -2 to -0.16], corresponding to an improvement in symptoms above the Mean Clinically Important Difference (MCID) threshold of -0.5. Blood was donated for flow cytometric analysis at the time points indicated in Figure E1. Blood was donated either one (-1) or 2 (-2) weeks prior to commencement of mepolizumab and then again on the day of 1^st^ injection (Figure E1) to constitute baseline measurements. One blood sample was then collected ~16 weeks later. 16-week follow-up blood samples were not obtained from 2/26 patients. In one patient, this was because of an adverse reaction that led to discontinuation of treatment, which was decided by our multi-disciplinary clinical team. A further patient declined consent for further research blood samples to be collected. The data from these two patients were excluded from the study. In the absence of placebo we also recruited 15 healthy subjects, not scheduled to receive mepolizumab, for consent, clinical assessment and blood collection as described above (and shown in Table E1).

**Flow Cytometry and ADVIA 2120/2120i**

Multi-parameter flow cytometric analysis was performed on 100µl of EDTA whole blood utilising a cocktail comprising CD193 VioBlue (Miltenyi Biotec) or, in some cases (n=5 blood samples), CD193 BV421 and CD4 BV510, CD69 BV605, CD123 BV711, Lineage (CD2, 3,14,16,56,235a) cocktail (Thermofisher), CD3 PerCP-Cy5.5, CD125 PE (Becton Dickinson), CD161 PE-Dazzle, Siglec-8 PE-Cy7, CD294-AF647, CD8 AF700, CD45 APC-Fire750 pre-mixed in Brilliant Violet stain buffer (all Biolegend unless otherwise stated). The same lot numbers were used throughout. Blood was incubated at room temperature for 15 mins prior to cell fixation and erythrocyte lysis (One step fix/Lyse, Thermofisher) according to the manufacturer’s instructions. Cells were re-suspended in 1ml PBS/2%FBS prior to acquisition of a fixed volume (900µl) on an Attune NxT acoustic focussing cytometer with compensation set-up using Ultracomp beads (Thermofisher). On each day of use, a performance check was performed prior to data collection and in all cases the Attune passed. Data were analysed using FlowJo software V10 (Tree Star, Oregon). Unconjugated CD294, Siglec-8, CD123 and CD69 antibodies were utilised to facilitate gating (CD294) or cell identification (Siglec-8, CD123). A CD161 FMO was incorporated into each run for comparison. After excluding doublets and CD45 negative cells, we measured the frequency and concentration of CRTH2 (CD294) positive eosinophils, Basophils, conventional (c)TH2, pathogenic effector (pe)TH2(1, 2), conventional (c)TC2 and ILC2s from baseline samples and following 16-weeks (Figure E2). The definition of each subset used for identification is given in the legend of Figure E2. For Eosinophils and Basophils we also measured cell surface expression of CD69, CD123 (Figure E2), CD125 (IL-5Rα), Siglec-8 and CD294 (GMFI).

From the same EDTA blood bottle, full blood count measurements were requested from the Blood Sciences Department in one of the UKAS accredited Leicester Pathology Service laboratories, which utilises the ADVIA 2120/2120i analyser (Siemens, Surrey, UK). The ADVIA 2120/2120i analyser performance is monitored in accordance with in-house and UKAS compliant quality assurance procedures and operated according to the manufacturer’s guidelines. The ADVIA 2120/2120i analyser utilises the peroxidase method to measure the frequency of eosinophils, as well as other cell types such as neutrophils, for which it shows good specificity (3, 4). This instrumentation, however, has poor specificity and sensitivity for basophils(4, 5). Notably in the clinical studies used to detect basophils, described in this letter, the authors have either not stated the instrumentation(6, 7) or used an ADVIA 120(8) or an H3(9) analyser.

In brief, Erythrocyte lysis and leucocyte fixation occurs using ‘Perox reagent 1’ (containing sodium dodecyl sulfate and Brij-35 and formaldehyde). Fixed Leucocytes were then stained using ‘Perox 2’ (4-chloro 1-naphthol) and hydrogen peroxide to form a dark precipitate. Cells were then visualised on a cell size vs absorption bivariate plot with eosinophils displaying a low cell size and high absorption (due to their eosinophil peroxidase content). Basophils are identified from their ability to resist lysis when diluted in ADVIA BASO reagent. In brief, erythrocytes and platelets were lysed in phthalic acid and surfactant. All white blood cells except basophils are stripped of their cytosplasm by the combined action of acid treatment and temperature (32-34̊C) in the reaction chamber. The intact basophils can be easily distinguished from the smaller cell nuclei by passing through a flow cell where, like flow cytometry, the low-angle light scatter (Y axis) and high-angle scatter (X axis) signatures of each cell are measured and plotted. The concentration of eosinophils and basophils (x10^9^/L) reported by the pathology service are based on a multiplication of cell frequency and the total leucocyte count (x10^9^/L). The data reported by the pathology service (x10^9^/L) were converted into the same units as the flow cytometry data (cells per 100µl) for comparison. The basophil frequency is not routinely reported. One follow-up value from a healthy subject was unavailable and thus the number of paired data sets from the ADVIA was 38 rather than 39.

For patients where we collected two bloods at baseline (2 or 1 week before injection and day of 1^st^ injection), the respective flow cytometric and ADVIA 2120/2120i measurements were averaged to constitute a single baseline value for comparison with the 16-week follow up data. In our healthy control group the flow cytometry data were treated in exactly the same way, however, only one of the baseline samples was sent to the pathology service for a full blood count measurement on their ADVIA 2120/2120i.

**Figure Legends**

**Figure 1**

Eosinophil and Basophils were measured per 100µl blood using flow cytometry (**A** and **B,** left) and on an ADVIA 2120/2120i analyser (**A** and **B,** right). In the asthma group, the eosinophil and basophil concentration was measured Pre and 16-weeks post, mepolizumab. In the healthy group, the eosinophil and basophil concentration was measured at baseline and 16-weeks later. Within group comparisons were made with a Wilcoxon Matched-Pairs test or a paired T-test (Healthy group, A, right and B, left). Dashed line indicates ADVIA 2120/2120i upper reference range.

**Figure 2**

**(A)** Eosinophil, **(B)** cTH2 and **(C)** peTH2 concentration pre Mepolizumab (y axis) plotted with the absolute change in ACQ6 score between baseline and 16-weeks post Mepolizumab (ΔACQ6, *x* axis). Linear regression (A, B) or Spearman (C) analysis was performed. Dashed lines on x axis represent the minimal clinically important difference (±0.5).

**Figure E1**

Study visit structure for Asthmatic patients (n=24) showing each mepolizumab dose every 4 weeks. Blood was collected one (-1) or two weeks (-2) before the 1^st^ dose of mepolizumab, on the day of the 1^st^ dose and on the day of the 5^th^ dose (16-week follow up). For comparison, blood was collected from non-asthmatic healthy volunteers (n=15) with the same time interval but without mepolizumab.

**Figure E2**

Flow Cytometry analysis depicted in pseudocolour dot plots showing **(A)** identification of basophils and eosinophil utilising combinatorial expression of CD123, CD294 and CD193. **(B)** Basophil and eosinophil events from the Boolean gate are shown from 1 asthmatic patient on the day of injection (red) and following 4^th^ dose of mepolizumab (blue). Note the reduced CD123 expression on eosinophils (987 vs 512, -48%) compared to basophils (28,012 vs 24,035, -14%). **(C)** CD3+ CD4+ or CD8+ T cells are depicted and **(D)** projected onto separate CD161 vs CD294 bivariate plots as indicated. Conventional CD161- CD294+ CD4+ cTH2 or CD8+ TC2 cells were identified by comparison to CD294- cells within the same data file and verified utilising an unconjugated CD294 antibody (“CD294 block”) and CD161 FMO as shown. peTH2 cells were defined as CD3+CD4+CD161+CD294+. **(E)** Lymphocyte gated, CD3- events were projected onto a bivariate dot plot displaying the lineage (Lin) cocktail and CD294 to identify Lin- CD294+ events consistent with the definition of ILC2s (and basophils). **(F)** ILC2s were defined as Lin-CD294+CD123-CD161+ (depicted as large dots) with tight light scatter properties (not shown). For each population the concentration (per 100µl of whole blood) and frequency were calculated. 100% of events are shown in each plot.

**Figure E3**

(**A**) Total white cell count (**B**) eosinophil concentration and (**C**) basophil concentration were recorded for asthmatic (filled circles) and healthy (empty squares) donors at baseline by Flow Cytometry (x-axis) and on an ADVIA 2120/2120i analyser (y-axis). Linear regression analysis was performed for each comparison. Dashed line indicates ADVIA 2120/2120i upper reference range.

**Figure E4**

Eosinophil (**A**) and basophil (**B**) frequency (of total CD45+ cells) was calculated using flow cytometry. Within group comparisons were made with a Wilcoxon Matched-Pairs test or a paired T-test (B, Healthy).

**Figure E5**

The baseline concentration of (**A**) eosinophils and (**B**) basophils (y-axis) were measured by flow cytometry and plotted against the difference (x-axis) between the 16-week and baseline measurements from patients receiving mepolizumab. As expected, in (**A**) with the exception of a few patients, those patients with the highest eosinophil counts at baseline respond to mepolizumab with a larger reduction in circulating levels of eosinophils at 16 week follow up. In (**B**) there was no consistent effect of mepolizumab in those with high or low basophil levels at baseline. Linear regression analysis was performed for each comparison.

**Figure E6**

Flow Cytometric measurement of (**A**) cTH2, (**B**) peTH2, (**C**) TC2 and (**D**) ILC2 concentration (left) and frequency (right, not calculated for ILC2s). Within group comparisons were made with a Wilcoxon Matched pairs test except in A cTH2 concentration, healthy; B peTH2 frequency, healthy and TC2 frequency, healthy (all Paired T-test).

**Figure E7**

**(A)** CD123 GMFI and **(B)** CD294 GMFI expression levels on eosinophils (left) and basophils (right) were measured at the same time intervals as Figure 1. Within group comparisons were made with either a Paired T-test or a Wilcoxon Matched-Pairs test (for CD123, A, Healthy, eosinophil and Asthma, basophil; for CD294, B, Asthma, basophil).

**Table E1 Subject clinical and biological demographics**

|  |  | Asthma  (*n*=24) | Healthy Controls^a^  (*n*=15) | p value |
| --- | --- | --- | --- | --- |
|  | Gender (M:F / M (%)) | 11:13 (46%) | 6:9 (40%) | 0.75 |
|  | Age (years±SD) | 53 ± 17 | 60 ± 7 | 0.08 |
|  | BMI (Kg/m^2^) | 33 ± 9 | 31±7 | 0.43 |
|  | Smoking status (Never, Ex-, Current) | 10, 14, 0 | 11,4,0 | 0.10 |
|  | Pack Year History^b^ | 0.63 [0-6] | 0 [0-2] | 0.16 |
|  | FEV_1_ (Litres), post BD | 2.20±0.75 | 2.80±0.69 | 0.02 |
|  | FEV_1_ (Litres) (%) | 74±20 | 104±11 | <0.0001 |
|  | FVC (Litres), post BD | 3.17±0.91 | 3.56±0.97 | 0.21 |
|  | FVC (%) | 88±18 | 107±13 | 0.0010 |
|  | FEV_1_ / FVC^b^ | 0.69 [0.63-0.79] | 0.79 [0.77-0.81] | 0.0011 |
|  | Total IgE (IU)^b^ | 79 [27-477] | 30 [13-123] | 0.043 |
| ADVIA 2120/2120i | Total WBC (per 100µl) | 1,085,208±269,248 | 567,333±108,921 | <0.0001 |
|  | Eosinophils (per 100µl)^b^ | 19,750 [11,250-41,250] | 15,000 [10,000-28,000] | 0.44 |
|  | Basophils (per 100µl)^b^ | 5,000 [4,125-7,000] | 4,000 [3,000-4,000] | 0.0084 |
| Attune NxT Flow Cytometry | Total CD45^+^ Singlets (per 100µl) | 533,572±157,102 | 382,311±69,729 | 0.0012 |
|  | Eosinophils (per 100µl)^b^ | 7,619 [2,968-14,561] | 8,203 [5,530-10,587] | 0.72 |
|  | Basophils (per 100µl) | 2,232±1,309 | 2,783±1,094 | 0.18 |
|  | cTH2 (per 100µl) | 633±547 | 648±330 | 0.93 |
|  | peTH2 (per 100µl)^b^ | 75 [54-190] | 59 [51-207] | 0.86 |
|  | TC2 (per 100µl)^b^ | 143 [88-319] | 253 [102-639] | 0.18 |
|  | ILC2s (per 100µl)^b^ | 14 [3-23] | 14 [7-28] | 0.68 |

BMI = body mass index; OCS = oral corticosteroid dose; ICS = Inhaled corticosteroid dose; ILC2 = Type-2 Innate lymphoid cell. ^a^Mepolizumab was not given to Healthy controls ^b^Median [IQR], all other values are mean ±SD unless stated.

**References**

1. Wambre E, DeLong JH, James EA, Torres-Chinn N, Pfutzner W, Mobs C, et al. Specific immunotherapy modifies allergen-specific CD4(+) T-cell responses in an epitope-dependent manner. *J Allergy Clin Immunol* 2014;**133**(3):872-879 e877.

2. Mitson-Salazar A, Prussin C. Pathogenic Effector Th2 Cells in Allergic Eosinophilic Inflammatory Disease. *Front Med (Lausanne)* 2017;**4**:165.

3. Amundsen EK, Urdal P, Hagve TA, Holthe MR, Henriksson CE. Absolute neutrophil counts from automated hematology instruments are accurate and precise even at very low levels. *Am J Clin Pathol* 2012;**137**(6):862-869.

4. Harris N, Jou JM, Devoto G, Lotz J, Pappas J, Wranovics D, et al. Performance evaluation of the ADVIA 2120 hematology analyzer: an international multicenter clinical trial. *Lab Hematol* 2005;**11**(1):62-70.

5. Amundsen EK, Henriksson CE, Holthe MR, Urdal P. Is the blood basophil count sufficiently precise, accurate, and specific?: three automated hematology instruments and flow cytometry compared. *Am J Clin Pathol* 2012;**137**(1):86-92.

6. Kelly EA, Esnault S, Liu LY, Evans MD, Johansson MW, Mathur S, et al. Mepolizumab Attenuates Airway Eosinophil Numbers, but Not Their Functional Phenotype, in Asthma. *Am J Respir Crit Care Med* 2017;**196**(11):1385-1395.

7. Nair P, Pizzichini MM, Kjarsgaard M, Inman MD, Efthimiadis A, Pizzichini E, et al. Mepolizumab for prednisone-dependent asthma with sputum eosinophilia. *N Engl J Med* 2009;**360**(10):985-993.

8. Flood-Page P, Menzies-Gow A, Phipps S, Ying S, Wangoo A, Ludwig MS, et al. Anti-IL-5 treatment reduces deposition of ECM proteins in the bronchial subepithelial basement membrane of mild atopic asthmatics. *J Clin Invest* 2003;**112**(7):1029-1036.

9. Buttner C, Lun A, Splettstoesser T, Kunkel G, Renz H. Monoclonal anti-interleukin-5 treatment suppresses eosinophil but not T-cell functions. *Eur Respir J* 2003;**21**(5):799-803.
